# Five Species of Wild Freshwater Sport Fish in Wisconsin, USA, Reveal Highly Diverse Viromes

**DOI:** 10.3390/pathogens13020150

**Published:** 2024-02-07

**Authors:** Charlotte E. Ford, Christopher D. Dunn, Eric M. Leis, Whitney A. Thiel, Tony L. Goldberg

**Affiliations:** 1 Department of Pathobiological Sciences, University of Wisconsin-Madison, Madison, WI 53706, USA; ceford4@wisc.edu (C.E.F.); cddunn2@wisc.edu (C.D.D.); 2 U.S. Fish and Wildlife Service, La Crosse Fish Health Center—Midwest Fisheries Center, Onalaska, WI 54650, USA; eric_leis@fws.gov; 3 Robert P. Hanson Laboratories, University of Wisconsin-Madison, Madison, WI 53706, USA; whitney.a.thiel@gmail.com

**Keywords:** freshwater fish, virome, sport fish, coronavirus

## Abstract

Studies of marine fish have revealed distant relatives of viruses important to global fish and animal health, but few such studies exist for freshwater fish. To investigate whether freshwater fish also host such viruses, we characterized the viromes of five wild species of freshwater fish in Wisconsin, USA: bluegill (*Lepomis macrochirus*), brown trout (*Salmo trutta*), lake sturgeon (*Acipenser fulvescens*), northern pike (*Esox lucius*), and walleye (*Sander vitreus*). We analyzed 103 blood serum samples collected during a state-wide survey from 2016 to 2020 and used a metagenomic approach for virus detection to identify known and previously uncharacterized virus sequences. We then characterized viruses phylogenetically and quantified prevalence, richness, and relative abundance for each virus. Within these viromes, we identified 19 viruses from 11 viral families: *Amnoonviridae*, *Circoviridae*, *Coronaviridae*, *Hepadnaviridae*, *Peribunyaviridae*, *Picobirnaviridae*, *Picornaviridae*, *Matonaviridae*, *Narnaviridae*, *Nudnaviridae*, and *Spinareoviridae*, 17 of which were previously undescribed. Among these viruses was the first fish-associated coronavirus from the *Gammacoronavirus* genus, which was present in 11/15 (73%) of *S. vitreus*. These results demonstrate that, similar to marine fish, freshwater fish also harbor diverse relatives of viruses important to the health of fish and other animals, although it currently remains unknown what effect, if any, the viruses we identified may have on fish health.

## 1. Introduction

Much of our current understanding of viruses infecting fish is based on the study of pathogenic viruses in symptomatic hosts. However, the recent rise of metagenomic sequencing has led to the discovery that fish harbor a greater number of viruses than any other class of vertebrates [1,2,3,4,5,6,7]. Most families of RNA viruses once thought to infect mammals have been described in bony fishes [1]. For example, a survey of dead/moribund chinook salmon (*Oncorhynchus tshawytscha*) revealed the first coronavirus (*Coronaviridae*) associated with fish, pacific salmon nidovirus [8]. Subsequent transcriptome analyses of publicly available sequence data identified additional distinct sub-families of coronaviruses in fish and amphibian transcriptomes [9,10]. Similarly, filoviruses (*Filoviridae*), previously thought to exclusively infect mammals, were discovered in European perch (*Perca fluviatilis*) and marine greenfin horse-faced filefish (*Thamnaconus septentrionalis*) [2,11]. Whilst these viruses retain a number of key features of other filoviruses, they are genetically distinct from the ebolaviruses and marburgviruses (renowned for causing lethal disease in humans) [12]. Likewise, hepadnaviruses, once known only from mammals and birds, infect amphibians and fish [13].

Despite rapid advances in our understanding of fish viromes, most knowledge to date has been obtained from studies of wild marine fish. Novel viruses from families known to infect marine fish [6,14], as well as families previously only associated with mammals, have been described [1,2]. One such study was Geoghegan et al. (2021), who identified the first fish-associated matonavirus (f. *Matonaviridae*), tiger flathead matonavirus, sharing only 28% amino acid identity (RDRP gene) with its famous relative, rubella virus, the causative agent of rubella in humans. They speculated that this family, as well as other viral families such as *Hantaviridae* and *Filoviridae*, may have originated in fish, evidenced by their basal phylogenetic positions [15]. They also hypothesized that vertebrate-associated viromes of fish were shaped by phylogenetic history of their hosts, with cross-species transmission also a common occurrence throughout virus evolution [15]. In general, it is clear that further examination of viral diversity in fish could deepen our understanding of the evolutionary origins of many viral families [16].

Viromes of wild freshwater fish are comparatively less studied than those of their marine counterparts. In most instances, such studies have been conducted in response to mortality events in wild fish [17,18] or in aquaculture settings [11]. We could only find a handful of broad virome surveys targeting wild freshwater fish, and none that had been conducted in the USA, to our knowledge [5,7,19,20,21]. This lack of data on wild freshwater fish viromes represents a potentially substantial knowledge gap. Lundberg et al. (2000) estimated that 10,000 fish species reside in freshwater bodies, making up 40% of all fish species [22]. Given this information, and the finding that the alpha diversity of vertebrate-associated viruses may be greater in freshwater fish than in their marine counterparts [5], studies examining viral diversity in wild freshwater fish would likely prove informative.

We conducted a study of the blood viromes of five species of sport fish across the state of Wisconsin, USA, collected from 2016 to 2020. Sport fishing in Wisconsin holds great cultural and economic significance [23], and viral diseases have previously impacted wild fish populations in the state, sometimes dramatically [18,24]. We performed a metagenomic survey of apparently healthy fish to (1) examine the total virome composition of these fish, (2) describe the phylogenetic relationships of potentially novel viruses, and (3) investigate whether viral prevalence, richness, or abundance varies between factors such as host species, location, or viral family. Our results support the notion that freshwater fish harbor highly diverse viromes, extending our knowledge of the geographical and host range over which such fish viruses occur.

## 2. Materials and Methods

### 2.1. Sampling Collection and Preparation

**Figure 1:**
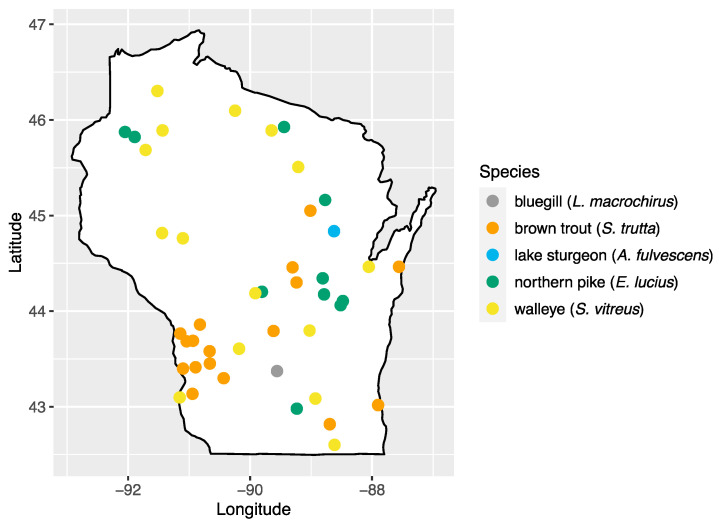
Map of sampling areas throughout Wisconsin where bluegill, brown trout, northern pike, lake sturgeon, and walleye were collected in this study.

We analyzed 103 serum samples collected from five species of fish during a previous study of viral hemorrhagic septicemia virus (*Rhabdoviridae: Novirhabdovirus*) [24]. Fish species were chosen based on their importance to sport fishing in the state: walleye (*Sander vitreus*; *n* = 17), bluegill (*Lepomis macrochirus*; *n* = 14), brown trout (*Salmo trutta*; *n* = 18), northern pike (*Esox lucius*; *n* = 23), and lake sturgeon (*Acipenser fulvescens*; *n* = 31). Sampling locations consisted of 43 inland waterbody sites across the state of Wisconsin, with multiple species present at 20 of these sites (Figure 1, Table S1). Samples were collected by the Wisconsin Department of Natural Resources as part of surveys of fish health across Wisconsin and were made available for this study [24]. Briefly, fish were captured using a number of methods (fyke netting, boom shocking, stream shocking, and capture via spawning weir), blood samples were collected from the caudal vein using an 18–22 gauge needle and 3–5 mL syringe and transferred to no-additive, red top glass blood tubes. Samples were stored at 4 °C to encourage clotting and, after 24 h, samples were centrifuged at 3200× *g* for 15 min, and the sera transferred to sterile 2 mL cryovials and stored at −80 °C until processing.

### 2.2. Next-Generation Sequencing

Metagenomic next-generation sequencing was performed as previously described [6,18,25,26]. In brief, samples were centrifuged at 10,000× *g* (4 °C) for 10 min to remove debris, then supernatants were transferred to a new tube and centrifuged further at 25,000× *g* (4 °C) for 3 h to pellet viruses. Nucleic acids were extracted from the resulting viral pellets using the QIAamp MinElute Virus Spin Kit (Qiagan, Germantown, MD, USA), and viral RNA was converted to double-stranded complementary DNA (cDNA) using the SuperScript IV First-Strand Synthesis System (Invitrogen, Waltham, MA, USA) and NEBNext^®^ Ultra™ II Non-Directional RNA Second Strand Synthesis (New England BioLabs, Ipswich, MA, USA). Samples then underwent purification using AmpureXP beads (Beckman Coulter, Indianapolis, IN, USA), and libraries were prepared using the Nextera XT DNA sample preparation kit (Illumina, San Diego, CA, USA) for sequencing on a MiSeq instrument using the MiSeq Reagent Kit, V3 chemistry, for 600 cycles (Illumina, USA).

### 2.3. Bioinformatic Analysis

Sequence data from all fish species were initially processed using *CLC Genomics Workbench v.23.0.2* (Qiagen). Sequence data were trimmed to remove low-quality (<Q30) and short-length (<50 bp) reads. For each fish species, sequences mapping to host DNA and known contaminants were removed and the remaining reads underwent de novo assembly using the metagenomic assembler *metaSPAdes v.3.15.5* [27], with resulting contiguous sequences (contigs) shorter than 500 bp discarded. Putative viral contigs were then queried against the NCBI non-redundant (nr) database at the protein level using *DIAMOND v.2.0.14.152* (blastx, --very-sensitive), with the subsequent *DIAMOND* alignment archive (DAA) file processed by *MEGAN v.6.25.3* to produce taxonomic assignments with reference to the NCBI taxonomy, using the lowest common ancestor (LCA) algorithm [28,29,30]. To confirm the putative viral contigs thus identified, we conducted homology searches against the NCBI nucleotide database (nt) and Conserved Domains Search Database [31].

Viruses were described based on their closest match in GenBank, with information on the natural hosts of each virus family identified from the International Committee on the Taxonomy of Viruses (ICTV) and ViralZone [32,33]. Viral families unlikely to infect fish (e.g., environmental viruses or viruses with non-vertebrate host ranges) were recorded but not analyzed further. For the resulting putative fish-infecting viruses, protein functions encoded by the sequence of each contig were inferred using a combination of the annotation software, *Cenote-taker2 v.2.1.2* [34], ORFfinder, and the NCBI Conserved Domains Search Database [35]. 

To calculate viral abundance, reads from each fish were mapped to each viral target sequence (length fraction = 1, similarity = 0.9). A virus was considered present if a fish had ≥2 overlapping viral reads of 50 bp or more. Reads that mapped to each virus, or any of the viruses examined (total viral load), were then normalized for sequencing depth and viral sequence length and log-transformed to become log10 viral reads per million per kilobase of target sequence (Log10vRPM/kb) [6,25,26].

### 2.4. Phylogenetic Analyses

Closely related amino acid sequences to the viral open-reading frames (ORFs) of interest were identified through a blastp search against GenBank [35]. These, in addition to member species of each viral family under investigation, were used in phylogenetic analysis of viral sequences identified as described above [36]. Amino acid sequences were aligned using *MUSCLE v.3.8*, and multiple codon alignments were constructed from the aligned proteins using *PAL2NAL v.14* [37,38]. The codon alignment was then trimmed using *trimAL v.1.4.1* (default settings), with minor manual adjustments made as needed. A suitable nucleotide evolutionary model was selected for each alignment using *ModelTest-NG* and a Bayesian approach to inferring phylogenetic relationships was performed using *MrBayes v.3.2.7* (ngen = 100,000, samplefreq = 500, burninfrac = 0.25) implemented in *NGPhylogeny* [39,40,41]. The resulting trees with posterior probability values were rooted at the midpoint and visualized using *Figtree v.1.4.4* [42].

### 2.5. Statistical Analysis

The proportion of positive individuals for each virus was used to indicate viral prevalence, with 95% confidence intervals calculated using the modified Wald method [43]. We compared prevalence, richness (number of viruses per fish), and normalized abundance among species, location (region of Wisconsin), and viral family using Kruskal–Wallis tests (*Kruskal.test*) and Wilcoxon rank-sum tests with Benjamini–Hochberg adjustment for pairwise comparisons (*pairwise.wilcox.test, p.adjest.method* = “*BH*”; [44]) in R studio v.1.2.5 [45].

## 3. Results

A total of 7552 contigs were assembled from all fish samples; 1753 from walleye, 1262 from bluegill, 197 from brown trout, 3700 from northern pike, and 640 from lake sturgeon. Of these contigs, 42 were viral in origin, 17 of which were thought to have originated from environmental contamination and so were not analyzed further (Table S2). The remaining 23 sequences represented viruses comprising five Baltimore classes: II (ssDNA), III (dsRNA), IV (ssRNA[+]), V (ssRNA[-]), and VII (dsDNA-RT). These sequences were most closely related to known viruses from 11 taxonomic groups, and one sequence most closely related to a currently unclassified known virus (Table 1). 

Viruses closely related to those originating from fish hosts or other vertebrates and thus likely infecting fish were analyzed further. This included 19 viruses in total, representing the following viral families: *Amnoonviridae*, *Circoviridae*, *Coronaviridae*, *Hepadnaviridae*, *Matonaviridae*, *Narnaviridae*, *Nudnaviridae*, *Peribunyaviridae*, *Picobirnaviridae*, *Picornaviridae*, and *Spinareoviridae*.

**Figure 2:**
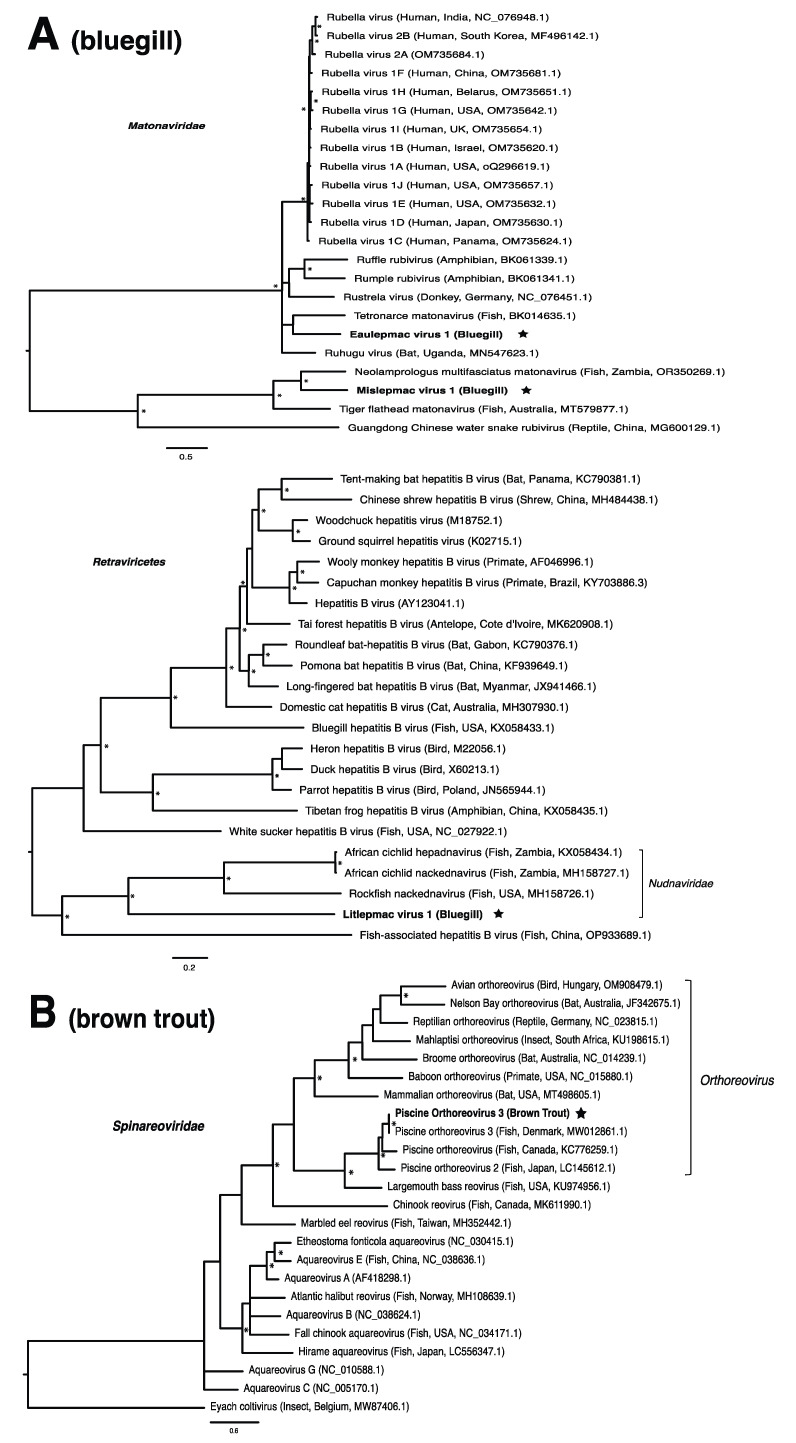

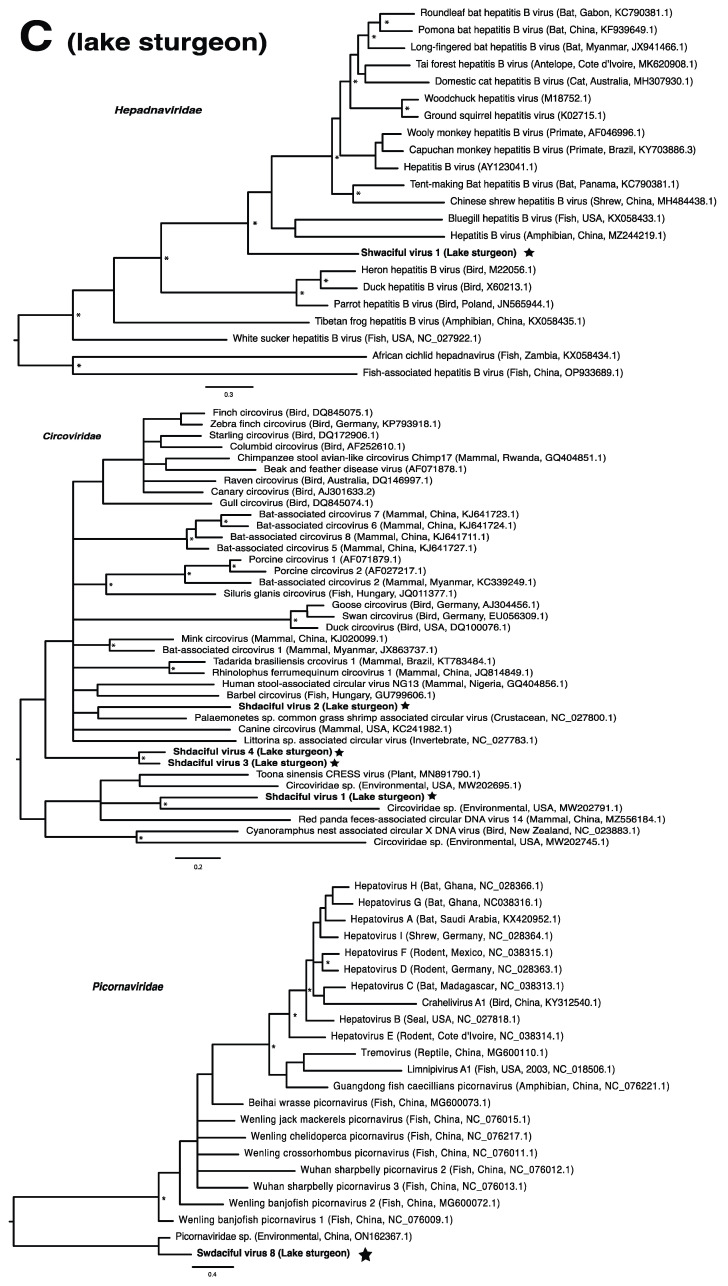

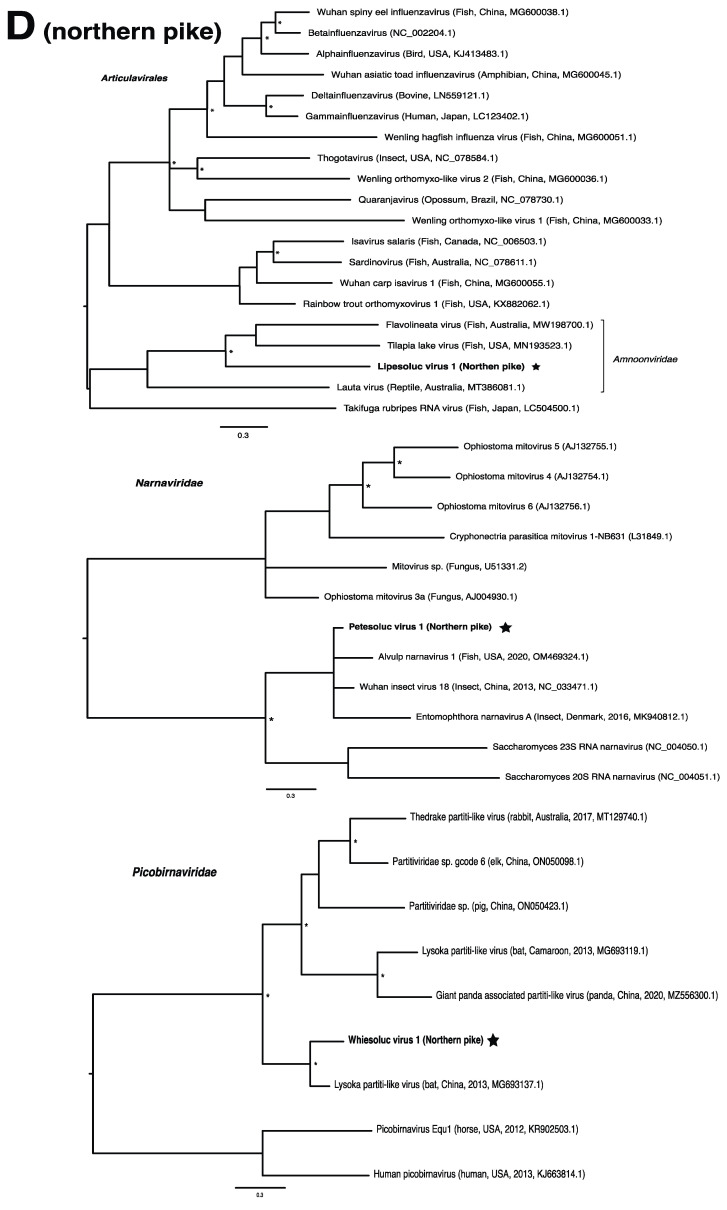

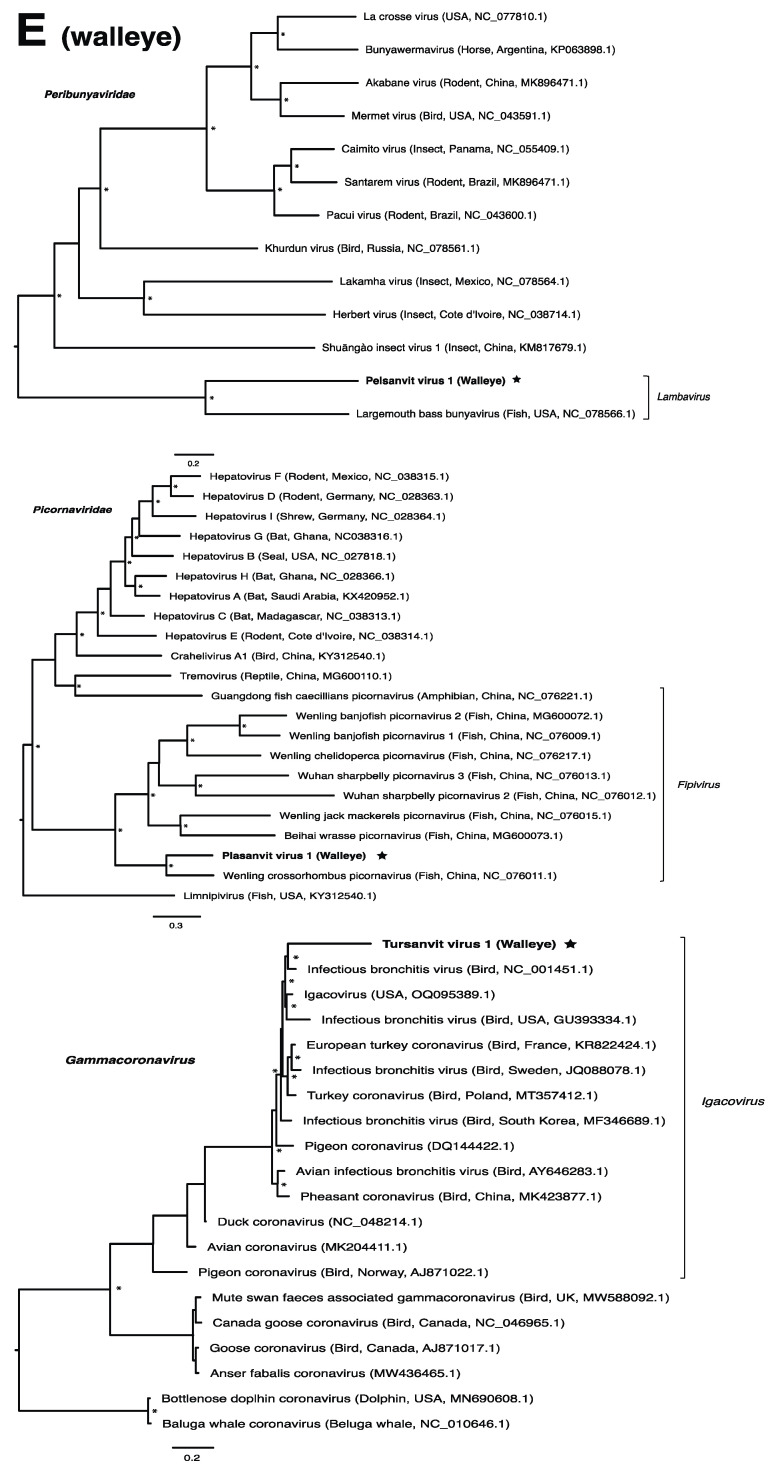
Bayesian inference of phylogenetic relationships for viruses in (**A**) bluegill (*L. macrochirus*), (**B**) brown trout (*S. trutta*), (**C**) lake sturgeon (*A. fulvescens*), (**D**) northern pike (*E. lucius*), (**E**) walleye (*S. vitreus*), and their relatives. Where available, virus names are followed by host, country, and GenBank accession number in parentheses. * indicates posterior probabilities ≥95%. Scale bar = substitutions per site. ORFs and substitution models used for each tree are given in Table S4. Star next to virus name indicates viruses originating from this study.

These viruses showed approximately 25% to more than 95% amino acid identity with their closest matches in GenBank (Table 1). Two viral contigs contained complete open reading frames (ORFs), and the remainder contained partial ORFs (Table S3). Phylogenetic analyses showed that most viruses investigated, excluding some belonging to the families *Circoviridae*, *Coronaviridae*, *Picornaviridae* (lake sturgeon only), and *Picobirnaviridae*, were most closely related to viruses previously identified in fish (Figure 2). Of these, all but piscine orthoreovirus 3 are previously undescribed members of their taxonomic group. Within the genus *Gammacoronavirus* (f. *Coronaviridae*), phylogenetic analysis revealed the virus under investigation (designated Tursanvit virus 1 by the authors) to be most closely related (94% amino acid identity within the nucleocapsid region) to infectious bronchitis virus (IBV) [46], an avian coronavirus belonging to the *Igacovirus* subgenus, for which the natural hosts are birds [47].

Viruses from the family *Picornaviridae* were identified in both lake sturgeon and walleye (Table 2), which was the only family present in more than one species. Lake sturgeon, northern pike, and walleye contained the highest viral diversity among the fish species analyzed, spanning three viral taxonomic groups each (with northern pike containing a further unclassified virus), followed by bluegill (two) and brown trout (one) (Table 2). 

Prevalence of infection with any virus was highest in lake sturgeon (96.77%), followed by walleye (70.59%), bluegill (42.86%), northern pike (39.13%), and brown trout (5.56%). Total viral prevalence did not vary significantly among species (*p* = 0.406), virus family (*p* = 0.443), or region of Wisconsin (*p* = 0.391; determined using the Kruskal–Wallis test). Viruses with particularly high prevalence included the circoviruses, shdaciful virus 2, 3, and 4, present in lake sturgeon at 83.87% (26/31), 64.51% (20/31), and 80.65% (25/31), respectively, followed by tursanvit virus 1 (TURSV-1), a coronavirus (f. *Coronaviridae*: g. *Gammacoronavirus*) found in 11/17 (64.71%) of walleye examined. Prevalence values of remaining viruses ranged from around 5% to 39% (Table S4).

**Figure 3:**
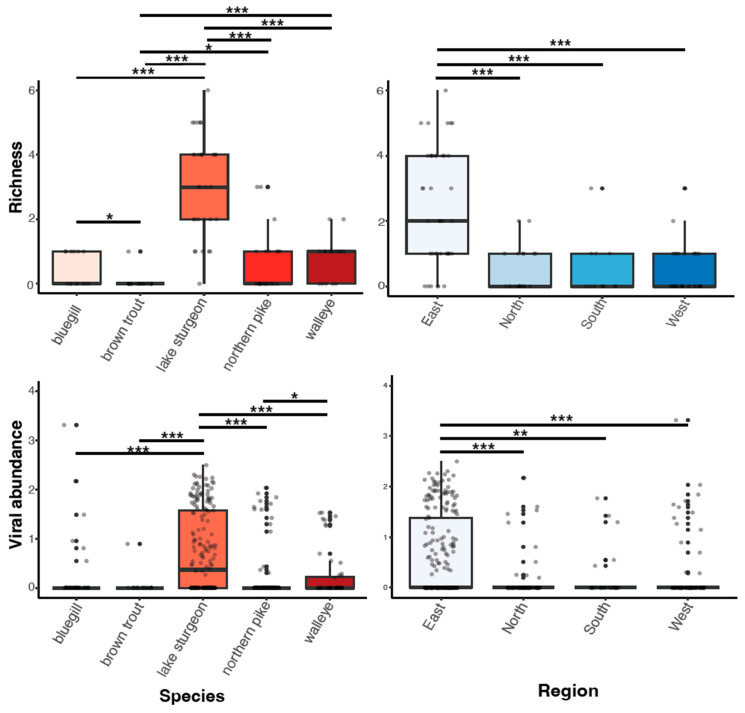
Boxplots of viral richness and viral abundance (log10vRPM/kb) for each species and regional location. *p* < 0.05(*), 0.01(**), 0.001(***) as determined by Wilcoxon rank-sum test with a Benjamini–Hochberg adjustment.

**Figure 4:**
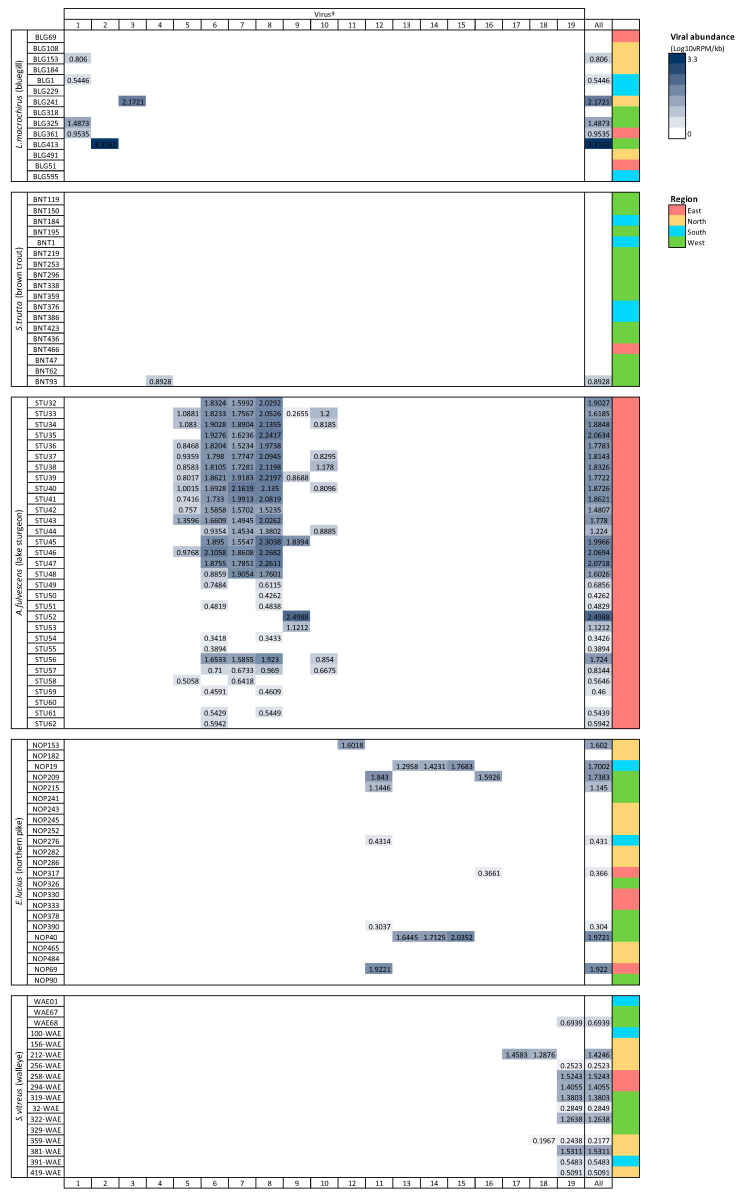
Heatmap of viral abundance across species of fish. Cells are shaded in proportion to the normalized abundance of each virus and total viral abundance for all viruses. † refers to ID in Table 1. Viral abundance unit (Log10vRPM/kb) = log10 viral reads per million per kilobase of target sequence.

Viral richness (number of viruses per fish) ranged from 0 to 6 and differed significantly among fish species (Kruskal–Wallis chi-squared = 60.55, df = 4, *p* ≤ 0.0001; Figure 3). This trend was predominantly driven by the significantly higher richness in lake sturgeon, and to a lesser extent, northern pike and walleye, when compared to the other species. Viral abundance also varied significantly among species (Kruskal–Wallis chi-squared = 78.08, df = 4, *p* ≤ 0.0001; Figure 3). Again, this was driven by high viral abundance in lake sturgeon. Sampling region had a significant effect on both viral abundance (Kruskal–Wallis chi-squared = 50.57, df = 3, *p* ≤ 0.0001) and richness (Kruskal–Wallis chi-squared = 36.79, df = 3, *p* ≤ 0.0001), though this may be due to the large sampling numbers from the eastern region of Wisconsin. The virome of each fish species was unique, in that no individual virus was shared among fish species (Figure 4).

## 4. Discussion

In this study, we described the blood virome of five freshwater species of sport fish found in Wisconsin, USA. We identified 19 likely fish-infecting viruses, 17 of which are novel, with closest known relatives spanning nine viral families (*Amnoonviridae, Circoviridae, Coronaviridae*, *Hepadnaviridae*, *Peribunyaviridae*, *Picobirnaviridae*, *Picornaviridae*, *Matonaviridae*, and *Nudnaviridae*). We also identified a previously undescribed virus of which the family was unassigned. 

### 4.1. Wisconsin Fish Harbor Diverse Novel Viruses

Wisconsin fish harbor viruses spanning five Baltimore classes (II, III, IV, V, VII), with RNA viruses most common and group IV predominant. This is not unexpected; there have been far more RNA virus families identified in fish than DNA viruses [2,48]. Although members of the families *Circoviridae*, *Coronaviridae*, *Hepadnaviridae*, *Picornaviridae*, and *Spinareoviridae* have also been found in previous metagenomic studies of wild freshwater fish [7,19,20,21], viruses from *Amnoonviridae*, *Matonaviridae*, *Narnaviridae*, *Nudnaviridae,* and *Peribunyaviridae* have, to our knowledge, not previously been described. Some members of these families have been detected in freshwater species previously, mostly through investigation of mortality events (e.g., tilapia lake virus; [49]), or by mining GenBank to study virus evolution [16]. 

Viruses in the *Picornaviridae* family were the only ones present in more than one species examined in this study. Picornaviruses are globally distributed and infect vertebrates of all classes. The family is extremely diverse, spanning 63 genera and 147 species [50,51]. Multiple picornaviruses have been associated with mortality in fish, causing hemorrhaging at the base of the fins and skin [17,52,53,54]. Whilst the majority of picornaviruses in the study are postulated to have originated from the environment, one virus, plasanvit virus 1 (PLASV-1), was discovered in walleye, of which the closest relative was Wenling crossorhombus picornavirus (pathogenicity unknown; [2]). 

We also found piscine orthoreovirus 3 (PRV-3; brown trout) belonging to the genus *Orthoreovirus* within the family *Spinareoviridae*. PRV-3, a segmented double-stranded DNA virus, is known for causing heart inflammation in rainbow trout [55], and is currently being debated as to its association with proliferative darkening syndrome in brown trout [56,57]. Although numerous disease outbreaks have been associated with PRV-3 [55,56,58,59], there have also been reports of asymptomatic infections in brown trout [60,61]. Asymptomatic infections are often responsible for the persistence and spread of pathogens [62,63]. For example, the non-virulent infectious salmon anemia virus (ISAV-HPR0), detected in Canada since 1998 in the absence of virulent strains, has also been implicated in disease outbreaks through the transition of ISAV-HPR0 to ISAV-HPRΔ, its virulent form [64,65]. Similarly, the survivors of an infectious pancreatic necrosis virus outbreak became carriers of the virus, allowing the virus to persist and spread, sometimes over years [66]. We note that largemouth bass reovirus, an orthoreovirus, was previously discovered in Wisconsin following a fish kill of largemouth bass (*Micropterus salmoides*; [18]). 

Members of the family *Amnoonviridae*, to which lipesoluc virus 1 in northern pike identified in this study putatively belongs, have also been associated with disease in fish. Tilapia lake virus, for instance, is the causative agent of a disease which induces lethargy, ocular alterations and skin erosion, and has resulted in mass mortalities of both wild and cultured Tilapia [67]. Together with flavolineata virus, which is not known to cause disease [68], tilapia lake virus is the known closest relative of LIPEV-1. Whether LIPEV-1 or the other aforementioned viruses cause disease in the fish in which we found them remains unknown.

We note that viruses from the families *Narnaviridae* and *Picobirnaviridae* may not directly infect their animal hosts, but rather the fungi and/or bacteria residing within them [69,70]. As members of the *Narnaviridae* family, for instance, infect a diverse array of hosts such as fungi, plants and protists, they may be useful indicators of other threats to fish health such as from endoparasites or disease-associated fungi [15,71]. We also note that viruses from the family *Circoviridae* were particularly abundant within this study, driven by lake sturgeon. Circoviruses have only recently been discovered in fish and amphibians, but it is becoming clear that circoviruses may be common in fish [72,73,74]. Whether these viruses are associated with disease in fish has yet to be determined.

### 4.2. Viromes of Wild Freshwater Fish Reveal Further Insights into Evolutionary Histories of Viral Families

Until recently, the only known species within the family *Matonaviridae* was rubella virus, a pathogen of global concern to human health [75,76]. More recently, this has been expanded to include matonaviruses in other mammals, as well as amphibians, reptiles, and fish [10,77,78,79,80]. In this study, we identified two putative matonaviruses in bluegill; the first, to our knowledge, to be identified in a freshwater species. Mislepmac virus 1 (MISLV-1) clustered with a distinct group of fish/reptile-associated matonaviruses, whereas eaulepmac virus 1 (EAULV-1) was most closely related to tetronarce matonavirus (TeMV) found in a Pacific eel ray (*Tetronarce*, *tetronarca californica*). It is intriguing that both MISLV-1 and EAULV-1, representing the two basally diverging lineages within the matonavirus phylogeny (Figure 2A), were found in bluegill in the same geographic location. This finding suggests that the evolutionary diversification of fish viruses may not depend on geographic separation, but rather the long-term virus–host co-divergence [1,9,21]. Our results are consistent with the notion that viruses from fish tend to form basally diverging lineages compared to viruses from other vertebrates, reflecting a longer evolutionary relationship with fish [2].

In the case of the hepadnaviruses, shwaciful virus 1 (SHWAV-1) is most closely related to an amphibian hepatitis B virus (sharing 40% amino acid identity), both of which form a group that is sister to the known hepadnaviruses of mammals, and more distantly related to known hepadnaviruses of non-mammalian vertebrates, including those of other fish (Figure 2C). This family is known to cause disease in a number of species. For instance, human-associated hepatitis B virus infects more than two billion people worldwide and can result in chronic liver disease and hepatocellular carcinoma in approximately 105 million people globally [81]. Similar pathology is observed in woodchuck and duck hepatitis B virus, both of which have been identified as causative agents of liver necrosis in their respective hosts [13]. However, to our knowledge, no disease has been associated with hepadnaviruses in fish. Unlike matonaviruses, hepadnaviruses identified from fish are found not just basally, but throughout the phylogenetic tree (Figure 2C). Interestingly, hepadnaviruses have occasionally experienced cross-species jumps over large evolutionary distances of hosts, as revealed through cophylogenetic reconciliation analyses [1], which may help to explain their phylogenetic positioning. Certainly, the discovery of additional members of the *Hepadnaviridae* will shed more light on the contributions of host switches to the evolutionary origins of what could have, until recently, been considered a family of mammalian specialist viruses. 

### 4.3. A Gammacoronavirus Detected in Walleye 

The family *Coronaviridae* contains enveloped, positive-strand RNA viruses with a broad host range. Most viruses within this family fall within the sub-family *Orthovirinae*, and predominantly infect mammals and birds, with the exception of a single virus from a reptilian host [2]. In recent years, the host range of this family has expanded to include another sub-family, *Letovirinae*, containing coronaviruses from both amphibian and fish hosts [8,82]. Mordecai et al. (2019) suggested that this sub-family was likely far larger than had been documented at the time [8], and indeed subsequent mining of transcriptomic data has revealed further viruses within this group. 

The genus *Gammacoronavirus* within the *Coronaviridae* family predominantly infects birds, although gammacoronaviruses have been found in aquatic mammals, forming the subgenus *Cegacovirus* [47,83]. The discovery of tursanvit virus 1 (TURSV-1) in walleye, represents (to our knowledge) the first such gammacoronavirus identified in fish. TURSV-1 is most closely related to infectious bronchitis virus (IBV), exemplar member of the subgenus *Igacovirus*, all of which infect birds [84,85]. IBV, predominantly a pathogen of chickens, affects the respiratory tract, gastrointestinal tract, kidney and reproductive systems of poultry, which has led to economic losses within the industry [46]. A fish-associated igacovirus would therefore significantly expand the host range of this subgenus. The replication ability and pathogenicity of TURSV-1 in walleye, however, remains unknown.

In summary, we identified a diverse assemblage of viruses in five wild freshwater species of fish in Wisconsin, USA. A number of these viruses are closely related to, or are known themselves to be, causative agents of disease in fish. Our virome profiles surely underestimate the full diversity of viruses in the fish we examined. Among other considerations, we examined viruses in blood, and research on wild marine fish has documented significant differences in virome composition among organs [4]. Nevertheless, our results show that freshwater fish host distant relatives of pathogens impacting global fish and animal health, including several viruses of importance to human health, which is a notion previously supported by research on marine fish. In addition to more studies of freshwater fish viromes in further geographic locations, we suggest that future research should focus on determining the pathogenicity of the viruses we have identified in fish and examining the genomes of novel viruses and their as-yet-undiscovered relatives in order to further examine the evolutionary histories of their respective families. 

## Figures and Tables

**Table 1:** Contigs representing viruses in Wisconsin sport fish.

Host	ID	Virus ^a^	Family	Natural Host(s) ^b^	Genome	Length	Cov.	Closest Match (Source, Location, Year, Accession) ^c^	E-Value ^c^	%ID (NR) ^b^	% Query Cov. ^c^
*Lepomis macrochirus* (bluegill)	1	Eaulepmac virus 1 (EAULV-1)	*Matonaviridae*	Vertebrates	ssRNA (+) [IV]	4317	327.1	Non-structural protein Rubella virus genotype 1E (human, Japan, 2018, BCT02655)	0	59.34	74
	2	Mislepmac virus 1 (MISLV-1)				8587	12.5	Non-structural protein Neolamprologus multifasciatus matonavirus (Fish, Zambia, 2016, WLN26226.1)	0	66.15	56
	3	Litlepmac virus 1 (LITLV-1)	*Nudnaviridae*	Fish	dsDNA-RT [VII]	2992	43.6	RDRP Paracyprichromis brieni nackednavirus (Fish, Burundi, 2015, WLN26319.1)	0	80.10	62
*Salmo trutta* (brown trout)	4	Piscine orthoreovirus 3 (PRV-3)	*Spinareoviridae*	Vertebrate/ Invertebrate/ Plant/ Fungi	dsRNA [III]	702	2	mu 2 protein Piscine orthoreovirus 3 (fish, Denmark, 2018, QOJ54106.1)	2 × 10^143^	99.51	99
						798	3.2	mu 1 protein Piscine orthoreovirus 3 (fish, Denmark, 2018, QOJ54098.1)	0	98.87	100
*Acipenser fulvescens* (lake sturgeon)	5	Shdaciful virus 1 (SHDAV-1)	*Circoviridae*	Birds/Mammals/ Fish	ssDNA [II]	537	2.6	Replication-associated protein Toona sinensis CRESS virus (Plant, QKI28974.1)	6 × 10^48^	45.35	96
	6	Shdaciful virus 2 (SHDAV-2)				1187	4.1	Replication-associated protein Palaemonetes sp. common grass shrimp associated circular virus (Shrimp, YP_009163936.1)	5 × 10^49^	38.03	59
**Host**	**ID**	**Virus ^a^**	**Family**	**Natural host(s) ^b^**	**Genome ^c^**	**Length**	**Cov.**	**Closest match (source, location, year, accession) ^d^**	**E-value ^c^**	**%ID (NR) ^d^**	**% Query cov. ^d^**
*Acipenser fulvescens* (lake sturgeon)	7	Shdaciful virus 3 (SHDAV-3)	*Circoviridae*	Birds/Mammals/ Fish	ssDNA [II]	563	4.7	Replication-associated protein *Littorina* sp. associated circular virus (Snail, YP_009163904.1)	2 × 10^26^	64.2	88
	8	Shdaciful virus 4 (SHDAV-4)				933	8.3	Replication-associated protein Cyanoramphus nest associated circular X DNA virus (Parakeet, New Zealand, 2012, YP_009021888.1)	2 × 10^70^	40.82	92
	9	Shwaciful virus 1 (SHWAV-1)	*Hepadnaviridae*	Humans/Apes/ Birds	dsDNA-RT [VII]	1831	47.5	DNA polymerase Hepatitis B virus (Amphibian, China, QWY26513.1)	7 × 10^111^	40.36	87
	10	Swdaciful virus 8	*Picornaviridae*	Vertebrates	ssRNA (+) [IV]	354	3.2	RNA Dependant RNA polymerase Picornaviridae sp. (environment, China, URG14974.1)	5 × 10^91^	64.1	99
*Esox lucius* (northern pike)	11	Lipesoluc virus 1 (LIPEV-1)	*Amnoonviridae*	Not available	ssRNA (−) [V]	1225	9.9	RNA Dependant RNA polymerase Flavolineata virus (fish, Australia, 2018, QPC41259.1)	6 × 10^68^	36.09	96
	12	Petesoluc virus 1	*Narnaviridae*	Fungi	ssRNA (+) [IV]	551	7.7	RNA Dependant RNA polymerase Alvulp narnavirus 1 (fish, USA, 2020, UVD33185.1)	2 × 10^110^	99.45	99
	13	Whiesoluc virus 5	*Picobirnaviridae*	Mammals/ Bacteria	dsRNA [III]	915	8.1	RNA Dependant RNA polymerase Lysoka partiti-like virus (bat, Cameroon, 2013, AWV67007.1)	0.00	81.91	99
	14	Whiesoluc virus 6				1646	8.2	Capsid protein Picobirnaviridae sp. (human, USA, 2015, DAH37469.1)	0.00	95.63	70
	15	Whiesoluc virus 7				839	20.1	Structural protein Picobirnaviridae sp. (environment, China, ULF99732.1)	0.00	72.13	60
	16	Petesoluc virus 2	*Unclassified*	Not available	ssDNA [II]	527	3.7	Putative viral replication protein Clictolig virus 1 (mussel, USA, 2018, UZT54550.1)	3 × 10^43^	41.95	99
**Host**	**ID**	**Virus ^a^**	**Family**	**Natural host(s) ^b^**	**Genome**	**Length**	**Cov.**	**Closest match (source, location, year, accession) ^c^**	**E-value ^c^**	**%ID (NR) ^c^**	**% Query cov. ^c^**
*Sander vitreus* (walleye)	17	Pelsanvit virus 1 (PELSV-1)	*Peribunyaviridae*	Reservoir: Rodents/ insects Vector: Ticks/mosquitoes Occasional: Human	ssRNA (−) [V]	5996	8.3	RNA Dependant RNA polymerase Largemouth bass bunyavirus (fish, USA, 2009, YP_010840272.1)	0	39.08	98
						1874	11.4	Glycoprotein Largemouth bass bunyavirus (fish, USA, 2009, YP_010840273.1)	3 × 10^32^	24.55	84
	18	Plasanvit virus 1 (PLASV-1)	*Picornaviridae*	Vertebrates	ssRNA (+) [IV]	1770	5.6	Polyprotein (RNA Dependant RNA polymerase) Wenling crossorhombus picornavirus (fish, China, YP_010796391)	0	63.61	99
						1016	7	Polyprotein (Capsid) Wenling crossorhombus picornavirus (fish, China, YP_010796391)	2 × 10^163^	69.94	99
						313	3.6	Polyprotein (Helicase) Wenling crossorhombus picornavirus (fish, China, YP_010796391)	1 × 10^28^	63.04	88
	19	Tursanvit virus 1 (TURSV-1)	*Coronaviridae*	Vertebrates	ssRNA (+) [IV]	1429	7.7	Nucleocapsid Infectious bronchitis virus (chicken, NP_040838.1)	0	93.9	85

^a^ As determined by phylogenetic analyses. All viruses have been named by the authors, except those previously described (PRV-3); ^b^ as described by the ICTV and/or ViralZone.expasy.org; ^c^ [] indicates the Baltimore classification for viruses; ^d^ closest match, E-value, and % identity (amino acid) were identified by querying viral contig sequences against NCBI’s non-redundant protein database using tblastx.

**Table 2:** Percentage viral prevalence in five wild fish species sampled from the Wisconsin fish survey (*L. macrochirus*, *S. trutta*, *A. fulvescens*, *E. Lucius*, and *S. vitreus*). Displayed is the proportion of individuals within each species that were positive for at least one virus from each viral taxonomic group. 95% confidence interval is shown in parentheses.

Family	Bluegill	Brown Trout	Lake Sturgeon	Northern Pike	Walleye
*Amnoonviridae*	0.00	0.00	0.00	4.35 (0.01–22.66)	0.00
*Circoviridae*	0.00	0.00	90.32 (74.31–97.44)	0.00	0.00
*Coronaviridae*	0.00	0.00	0.00	0.00	64.71 (41.16–82.83)
*Hepadnaviridae*	0.00	0.00	16.13 (0.00–22.66)	0.00	0.00
*Matonaviridae*	35.71 (16.18–61.40)	0.00	0.00	0.00	0.00
*Narnaviridae*	0.00	0.00	0.00	21.74 (9.23–42.33)	0.00
*Nudnaviridae*	7.14 (0.01–33.54)	0.00	0.00	0.00	0.00
*Peribunyaviridae*	0.00	0.00	0.00	0.00	11.76 (2.03–35.59)
*Picobirnaviridae*	0.00	0.00	0.00	8.7 (1.25–27.97)	0.00
*Picornaviridae*	0.00	0.00	25.81 (13.49–43.46)	0.00	5.88 (0.01–29.82)
*Spinareoviridae*	0.00	5.56 (0.01–27.65)	0.00	0.00	0.00
*Unclassified*	0.00	0.00	0.00	8.7 (1.25–27.97)	0.00
Total prevalence	42.86 (21.34–67.45)	5.56 (0.01–27.65)	96.77 (82.42–99.99)	39.13 (22.1–59.27)	70.59 (46.57–87.01)

## Data Availability

The sequence reads generated in this study are available at the NCBI Sequence Read Archive (SRA) database under BioProject accession PRJNA1053451. Viral sequences described in this study have been deposited in GenBank under accession numbers PP002545-PP002573. Details for individual fish sample locations, viruses described in this study, and phylogenetic information are provided in Supplementary Tables S1–S4.

## Supplementary Materials

The following supporting information can be downloaded at: https://www.mdpi.com/article/10.3390/pathogens13020150/s1, Table S1: Sampling locations for five fish species sampled during the Wisconsin fish survey; Table S2: Environmentally derived contigs present in samples; Table S3: ORFs and substitution models for each Bayesian phylogenetic tree; Table S4: Virus prevalence in each fish species.
