# Supplementary material for: Five Species of Wild Freshwater Sport Fish in Wisconsin, USA, Reveal Highly Diverse Viromes
Source: Pathogens. 2024 Feb 7;13(2):150. doi: 10.3390/pathogens13020150 (PMC10891596; doi:10.3390/pathogens13020150)
Supplement: Supplementary file 1 [file pathogens-13-00150-s001.zip › pathogens-2806027-supplementary.pdf]

Table S1: Sampling locations for the five fish species sampled during the Wisconsin fish survey (*L. macrochirus, S.trutta, A. fulvescens, E. Lucius* and *S. vitreus*).

**Water body**	**Latitude**	**Longitude**			**Species**		
Ash Creek	43.29906	-90.43173	-	-	-	Trout	-
Asylum Bay	44.06165	-88.51517	Bluegill	Pike	-	-	-
Besadny Anadromous Fisheries Area	44.46415	-87.5595	-	-	-	Trout	-
Bluff Creek	42.81745	-88.69301	-	-	-	Trout	-
Coon Valley	43.6844	-91.04043	-	-	-	Trout	-
Delevan Lake	42.60136	-88.6152	-	-	Walleye	-	-
Dutch Hollow Lake	43.60719	-90.17929	-	Pike	Walleye	-	-
Eau Claire Lake	44.76203	-91.10116	Bluegill	Pike	Walleye	-	-
Elk Creek	43.45217	-90.65797	-	-	-	Trout	-
Emmons Creek	44.30023	-89.23842	-	-	-	Trout	-
Fox River	44.4635	-88.05415	-	Pike	Walleye	-	-
Green Lake	43.79711	-89.02359	-	-	Walleye	-	-
Lac Courte Oreilles	45.89023	-91.43466	Bluegill	Pike	Walleye	-	-
Lake Altoona	44.81691	-91.44101		Pike	Walleye	-	-
Lake Kegonsa	42.97985	-89.23332	Bluegill	Pike	-	-	-
Lake Poygan	44.17544	-88.78686		Pike	-	-	-
Lake Sherwood	44.20257	-89.80482	Bluegill	Pike	-	-	-
Lake Winnebago	44.10607	-88.47873	Bluegill	Pike	-	-	-
Lake Wisconsin	43.37236	-89.55848	Bluegill	-	-	-	-
Lipsett Lake	45.87378	-92.04847	-	Pike	-	-	-
Little La Crosse River	43.85934	-90.8209	-	-	-	Trout	-
Little Saint Germaine Lake	45.92572	-89.4419	Bluegill	Pike	-	-	-
Long Lake	45.68567	-91.7106	-	Pike	Walleye	-	-
Madeline Lake	45.89	-89.64762	Bluegill	Pike	Walleye	-	-
Middle Eau Claire Lake	46.30306	-91.51782	Bluegill	Pike	Walleye	-	-
Milwaukee Harbour	43.01753	-87.90257	-	-	-	Trout	-
Mississippi River	43.09781	-91.1536	Bluegill	-	Walleye	-	-
Mormon Coulee	43.76548	-91.14373	-	-	-	Trout	-
Neenah Creek	43.7922	-89.61494	-	-	-	Trout	-
Nepco Lake	44.34373	-88.80911	Bluegill	Pike	-	-	-
Otter Creek	43.58098	-90.66241	-	-	-	Trout	-
Pelican Lake	45.50724	-89.21044	Bluegill	Pike	Walleye	-	-
Petenwell Lake	44.18712	-89.91348	-	Pike	Walleye	-	-
Plum Creek	43.135	-90.9445	-	-	-	Trout	-
Red River	45.05089	-89.01189	-	-	-	Trout	-
Rock Lake	43.08568	-88.92951	Bluegill	Pike	Walleye	-	-
Rush Creek	43.39904	-91.09828	-	-	-	Trout	-
Shawano Damn	44.8358	-88.62334	-	-	-	-	Sturgeon
Spring Coulee	43.69013	-90.93577	-	-	-	Trout	-
Tainter Creek	43.4132	-90.8926	-	-	-	Trout	-
Tomorrow River	44.4581	-89.30292	-	-	-	Trout	-
Turlte FlambeauFlowage	46.09615	-90.24521	-	Pike	Walleye	-	-
White Lake	45.16297	-88.76961	Bluegill	Pike	-	-	-
Yellow River	45.82126	-91.88982	-	Pike	-	-	-

Table S2: Environmentally-derived viral contigs present in samples from the five fish species examined in this study (*L. macrochirus, S.trutta, A. fulvescens, E. Lucius* and *S. vitreus*).

Host	Name	Length	Closest match (source, location, year, accession)[a]	E-Value[a]	% ID (NR)[a]
bluegill	Eaclepmac virus 1	695	Horse nettle virus A (plant, USA, 2021, WEY36561.1)	6.00E-92	69.7
	Asylepmac virus 1	511	Grapevine chrome mosaic virus (NP_619704.1)	4.00E-27	41.88
	Ecllepmac virus 1	413	Grapevine chrome mosaic virus (NP_619705.1)	7.00E-55	64.75
	Ecllepmac virus 2	632	Grapvine chrome mosaic virus (YP_002000610.1)	1.00E-93	63.81
lake sturgeon	Swdaciful virus 1	560	Otus toti-like virus (algae, DAZ87248.1)	8.00E-40	50
	Swdaciful virus 2	722	Red algae totivirus 1 (Japan, 2016, BBZ90082.1)	3.00E-59	50.87
	Swdaciful virus 3	370	Picornavirales sp. (enviroment, China, ULF99736.1)	2.00E-75	100
	Clinch densovirus 1	1342	Clinch densovirus 1 (mussel, USA, 2018, QNL09579.1)	5.00E-136	100
	Swdaciful virus 4	360	Clinch densovirus 1 (mussel, USA, 2018, YP_010802875)	3.00E-78	100
	Swdaciful virus 5	661	Sanxia water strider virus 8 (insect, China, 2013, YP_009337665.1)	2.00E-23	39.2
	Swdaciful virus 7	734	Dragonfly larvae associated circular virus-10 (insect, New Zealand, 2013, ALE29826.1)	3.00E-33	56.64
northern pike	Whiesoluc virus 1	8206	Novo Mesto dicistrovirus 2 (Plant, Slovenia, UTQ50718.1)	0	99.1
	Whiesoluc virus 2	3058	Novo Mesto dicistrovirus 2 (Plant, Slovenia, UTQ50718.1)	0	87.4
	Whiesoluc virus 3	1110	Dicistroviridae sp. (plant, China, ULF99799.1)	0	95.66
	Asyesoluc virus 1	545	Botourmiaviridae sp. (Fungus, Spain, 2018, UYL94570.1)	9.00E-121	95.58
	Whiesoluc virus 4	863	Plasmopara viticola lesion associated dicistro-like virus 1 (fungus, Italy, 2018, QHD64742.1)	2.00E-104	57.24
walleye	Gresanvit virus 1	304	Purpureocillium lilacinum nonsegmented virus 1 (fungus, 2016, AOO52900.1)	7.00E-23	99

[a] As described by the ICTV and/or ViralZone.expasy.org
[b] Closest match, E-value and % identity (amino acid) were identified by querying viral contig sequences against NCBI's non-redundant protein database using tblastx

Table S3: ORFs and substitution models used for each Bayesian phylogenetic tree

Species	Tree	ORF	ORF completedness	Substition model
Bluegill (*L.macrochirus*)	*Matonaviridae*	Non-structural protein containing RNA dependent RNA polymerase	Partial	GTR+G4
	Revtraviricetes	RNA dependent RNA polymerase	Complete	GTR+I+G4
Brown trout (*S.trutta*)	*Spinareoviridae*	mu2	Partial	GTR+I+G4
Lake sturgeon (*A.fulvescens*)	*Circoviridae*	Replication-associated protein	Partial	SYM+I+G4
	Hepadnaviridae	DNA polymerase	Partial	GTR+I+G4
	Picornaviridae	RNA dependent RNA polymerase	Partial	GTR+G4
Northern pike (E.lucius)	*Amnoonviridae*	RNA dependent RNA polymerase	Partial	GTR+I+G4
	Narnaviridae	RNA dependent RNA polymerase	Partial	GTR+I+G4
	Picobirnaviridae	RNA dependent RNA polymerase	Partial	GTR+G4
Walleye (*S.vitres*)	*Peribunyaviridae*	RNA dependent RNA polymerase	Partial	GTR+I+G4
	Picornaviridae	Polyprotein containing RNA dependent RNA polymerase	Partial	GTR+I+G4
	Gammacoronavirus	Nucleocapsid	Complete	GTR+I+G4

Table S4: Virus prevalence in each fish species (*L. macrochirus, S.trutta, A. fulvescens, E. Lucius* and *S. vitreus*).

Virus	bluegill	brown trout	lake sturgeon	northern pike	walleye
Eaulepmac virus 1	28.57	-	-	-	-
Mislepmac virus 1	7.14	-	-	-	-
Litlepmac virus 1	7.14	-	-	-	-
Piscine orthoreovirus 3	-	5.56	-	-	-
Shdaciful virus 1	-	-	38.71	-	-
Shdaciful virus 2	-	-	83.87	-	-
Shdaciful virus 3	-	-	64.51	-	-
Shdaciful virus 4	-	-	80.65	-	-
Shwaciful virus 1	-	-	16.13	-	-
Swdaciful virus 8	-	-	25.81	-	-
Lipesoluc virus 1	-	-	-	4.35	-
Petesoluc virus 1	-	-	-	21.74	-
Whiesoluc virus 5	-	-	-	8.7	-
Whiesoluc virus 6	-	-	-	8.7	-
Whiesoluc virus 7	-	-	-	8.7	-
Petesoluc virus 2	-	-	-	8.7	-
Pelsanvit virus 1	-	-	-	-	5.88
Plasanvit virus 1	-	-	-	-	11.76
Tursanvit virus 1	-	-	-	-	64.71
